# Association between the triglyceride–glucose index and subclinical atherosclerosis in asymptomatic adults: a large cross-sectional study of a health check-up population

**DOI:** 10.3389/fcvm.2026.1795702

**Published:** 2026-06-10

**Authors:** Zhihong Gao, Yuqiang Zuo, Yuling Yin, Xu Yang, Lei Xu, Min Jia, Lan Yang

**Affiliations:** 1 Department of Physical Examination Center, The Second Hospital of Hebei Medical University, Shijiazhuang, Hebei, China; 2 Department of Cardiology, Shijiazhuang People's Hospital, Shijiazhuang, Hebei, China; 3 Department of Cardiology, The Second Hospital of Hebei Medical University, Shijiazhuang, Hebei, China; 4 Department of Neurology, The Second Hospital of Hebei Medical University, Shijiazhuang, Hebei, China

**Keywords:** asymptomatic adults, insulin resistance, restricted cubic spline, subclinical atherosclerosis, TyG index

## Abstract

**Background:**

This study aimed to investigate the association between the triglyceride–glucose (TyG) index and subclinical atherosclerosis (SA), hypothesizing that higher TyG levels are associated with increased odds of SA and may exhibit a non-linear dose–response relationship.

**Methods:**

We conducted a cross-sectional study of 13,473 asymptomatic adults undergoing health examinations. SA was assessed by carotid ultrasonography. TyG was calculated from fasting triglyceride and glucose levels. Logistic regression and restricted cubic spline (RCS) models were used to evaluated the associations between TyG and SA.

**Results:**

Higher TyG levels were associated with increased odds of SA in a dose–response pattern. In fully adjusted models, each 1-unit increase in TyG was associated with 20% higher odds of SA (OR 1.20, 95% CI 1.10–1.30). Participants in the highest tertile had increased odds compared with the lowest tertile (OR 1.38, 95% CI 1.22–1.56; *P_trend_* <0.001). RCS analysis suggested a significant non-linear association (*P_non−linearity_* = 0.034), with an exploratory model-derived inflection point around 8.07. The association remained consistent in normoglycemic individuals (OR = 1.21; 95% CI: 1.02–1.44).

**Conclusions:**

In this large asymptomatic population, the TyG index showed a robust positive association with SA. These findings extend existing evidence by providing preliminary insights into the dose–response relationship, although the non-linear pattern should be interpreted with caution and requires further validation.

## Introduction

Cardiovascular disease (CVD) remains the leading cause of mortality worldwide, accounting for more than one third of global deaths and imposing a substantial burden on public health systems (1). Atherosclerosis, the fundamental pathological substrate of most cardiovascular events, typically progresses silently over decades before manifesting as acute clinical conditions such as myocardial infarction or stroke (2). Consequently, the early identification of individuals with subclinical atherosclerosis (SA) has become a critical priority in contemporary strategies cardiovascular prevention (3).

Insulin resistance (IR) plays a pivotal role in the initiation and progression of atherosclerosis through multiple interconnected pathways, including endothelial dysfunction, chronic low-grade inflammation, oxidative stress, and dysregulated lipid metabolism (4). Although the hyperinsulinemic–euglycemic clamp is considered the reference standard for assessing IR, its technical complexity and limited feasibility restrict its application in large-scale epidemiological studies and routine clinical practice (5). In this context, the triglyceride–glucose (TyG) index, derived from fasting triglyceride and glucose concentrations, has emerged as a simple, inexpensive, and reproducible surrogate marker of IR (6).

Accumulating evidence indicates that the TyG index is closely associated with a broad spectrum of cardiometabolic disorders, including hypertension, type 2 diabetes, and metabolic syndrome, as well as with markers of subclinical vascular injury such as coronary artery calcification and increased carotid intima–media thickness (7–9). Moreover, several cohort and cross-sectional studies have reported that elevated TyG levels are independently associated with higher risks of cardiovascular events and all-cause mortality (10).

Despite these advances, important gaps remain. Most previous investigations have focused on populations with established metabolic abnormalities or overt CVD, whereas data from apparently healthy, asymptomatic individuals are still limited. In such populations, traditional cardiovascular risk scores may underestimate the burden of early atherosclerotic changes. Furthermore, the dose-response relationship between the TyG index and SA has not been adequately characterized in large asymptomatic cohorts, and potential non-linear associations or threshold effects have rarely been explored.

In addition, existing studies have reported inconsistent findings regarding the modifying effects of sex, age, and obesity status on the TyG–atherosclerosis association, suggesting that comprehensive subgroup analyses may be warranted to better define vulnerable populations and refine risk stratification accuracy (11–13).

Therefore, the present study aimed to investigate the association between the TyG index and SA in a large health check-up population of asymptomatic adults. Multivariable logistic regression and restricted cubic spline (RCS) were performed to explore the dose–response relationship and assess potential non-linear patterns. Subgroup analyses were additionally conducted across major demographic and clinical strata.

## Materials and methods

**Figure 1:**
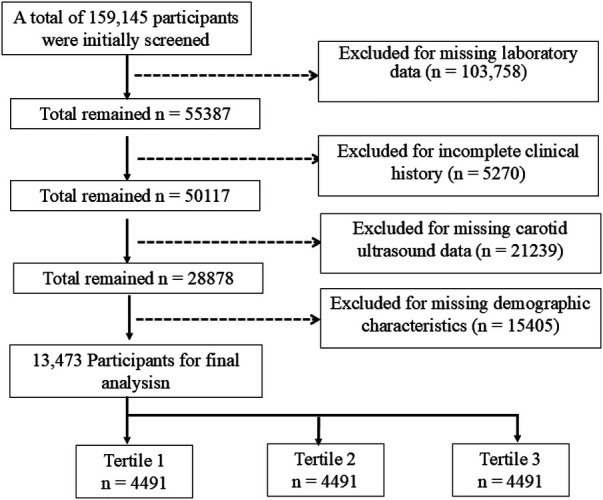
Flowchart of participant selection.

This cross-sectional study enrolled individuals who underwent routine health check-ups at the Physical Examination Center of the Second Hospital of Hebei Medical University between January 2021 and December 2024. Among 159,145 participants initially screened, a total of 13,473 participants met the eligibility criteria and were included in the final analysis (Figure 1). Included and excluded participants were generally comparable across baseline characteristics, suggesting a low risk of selection bias (Supplementary Table S1).

### Inclusion and exclusion criteria

Inclusion criteria were as follows: ① age ≥ 18 years; ② completion of routine physical examination with available demographic, anthropometric, biochemical, and carotid ultrasound data; ③ no prior history of cardiovascular events, including myocardial infarction, stroke, coronary revascularization, or peripheral arterial disease.

Exclusion criteria include: ① missing key clinical, laboratory, or imaging data; ② previous diagnosis of coronary artery disease, CVD, or peripheral arterial disease; ③ active infection, autoimmune disease, malignancy, or severe hepatic or renal dysfunction; ④ poor-quality carotid ultrasound images precluding reliable assessment.

### Data collection

Clinical data were extracted from the hospital electronic health record system.

Demographic and anthropometric variables included sex, age, height, weight, body mass index (BMI), systolic blood pressure (SBP), and diastolic blood pressure (DBP). BMI was calculated as weight (kg) divided by height squared (m^2^) and categorized according to Chinese adult BMI criteria as normal weight (< 24 kg/m^2^) and overweight/obese (≥ 24 kg/m^2^) (14). Blood pressure was measured using an automated sphygmomanometer after at least 5 min of seated rest.

Medical history, including physician-diagnosed hypertension, diabetes, and dyslipidemia, as well as lifestyle factors (smoking and drinking status), was obtained through standardized questionnaires. Current smoking was defined as smoking at least one cigarette per day for over one year. Regular drinking was defined as consumption of approximately 100 mL of Chinese spirits (alcohol content ≈ 50%) per day for more than one year.

### Laboratory measurements

All blood samples were collected after an overnight fast of at least 8 h. Venous blood samples were drawn between 7:00 and 9:00 AM and analyzed in the hospital central laboratory using standardized procedures on a Roche cobas 8000 automatic biochemical analyzer (Roche Diagnostics, Switzerland).

Measured parameters included complete blood count indices, high-sensitivity C-reactive protein (hs-CRP), total cholesterol (TC), triglycerides (TG), high-density lipoprotein cholesterol (HDL-C), low-density lipoprotein cholesterol (LDL-C), fasting plasma glucose (FPG), and uric acid (UA). The TyG index was calculated as ln [(TG (mg/dl) × FBG (mg/dl)/2].

### Definition of hypertension, dyslipidemia and diabetes

Hypertension was defined as self-reported physician-diagnosed hypertension, current use of antihypertensive medication, or SBP ≥ 140 mmHg and/or DBP ≥ 90 mmHg at baseline, according to the Seventh Report of the Joint National Committee (15).

Dyslipidemia was defined as self-reported history of dyslipidemia, use of lipid-lowering medication, or the presence of any of the following: TC ≥ 5.2 mmol/L, TG ≥1.7 mmol/L, LDL-C ≥ 3.4 mmol/L, or HDL-C < 1.0 mmol/L. Diabetes mellitus (DM) was defined as self-reported physician-diagnosed diabetes, current use of hypoglycemic medication, or FPG ≥ 7.0 mmol/L; impaired fasting glucose was defined as FPG ≥ 5.6 mmol/L but < 7.0 mmol/L, according to the American Diabetes Association 2024 criteria (16).

### Definition of SA

Carotid ultrasonography was performed by trained and experienced sonographers using a standardized imaging protocol. All examinations were conducted with a high-resolution Vivid 7 ultrasound system (GE Medical Systems, Milwaukee, WI, USA) equipped with a 7–12 MHz linear-array transducer. All ultrasound images were acquired during the end-diastolic phase. Carotid intima-media thickness (IMT) was defined as the distance between the lumen–intima interface and the media–adventitia interface. Measurements were performed on the near and far walls of the bilateral common carotid arteries and carotid bifurcations. The mean of maximum IMT value from these sites was used for subsequent analysis.

SA was defined as meeting either of the following criteria: (1) carotid intima–media thickness (CIMT) ≥ 1.0 mm; or (2) presence of a focal atherosclerotic plaque, defined as a focal structure encroaching into the arterial lumen by ≥ 0.5 mm or 50% of the surrounding IMT, or with a thickness > 1.5 mm measured from the media–adventitia interface to the intima–lumen interface (17).

### Statistical analysis

All statistical analyses were performed using R software (version 4.3.2; R Foundation for Statistical Computing, Vienna, Austria).

Continuous variables were expressed as mean ± standard deviation (SD) or median (Q1, Q3), as appropriate, and categorical variables as frequencies and percentages. Between-group comparisons were conducted using Student's t-test or Mann–Whitney U test for continuous variables and chi-square test for categorical variables.

Multivariable logistic regression assessed the association between TyG index (as a continuous variable and in tertiles) and SA across three progressively adjusted models (Model 1 was unadjusted; Model 2 was adjusted for age and sex; and Model 3 was further adjusted for BMI, smoking, SBP, HDL-C, and drinking based on Model 2). Multicollinearity among variables included in the multivariable models was assessed using variance inflation factors (VIF). A VIF > 5 was considered indicative of significant collinearity. Tests for trend were performed by assigning the median value of each tertile as a continuous variable.

RCS analysis was performed within a multivariable logistic regression framework to explore potential non-linear associations between TyG index and SA. Four knots were placed at the 5th, 35th, 65th, and 95th percentiles of the TyG distribution, following commonly recommended practices. The median value of TyG was used as the reference. RCS models were adjusted for the same covariates as in Model 3 (age, sex, BMI, smoking status, SBP, HDL-C, and drinking status). Odds ratios (ORs) and 95% confidence intervals (CIs) were derived from the fitted models to visualize the dose–response relationship. The overall association and non-linearity were assessed using likelihood ratio test by comparing models with and without spline terms. Sensitivity analyses were conducted by excluding participants with extreme TyG index values (outside the 2.5th–97.5th percentile range) to evaluate the impact of potential outliers on the RCS curve and the identified inflection point.

Subgroup analyses were conducted stratified by sex, age, BMI, SBP, and FPG, with FPG categorized as normoglycemic (FPG < 5.6 mmol/L) and elevated (FPG ≥ 5.6 mmol/L) based on standard clinical thresholds. In each subgroup, multivariable logistic regression models were applied, adjusted for age, sex, BMI, smoking, SBP, HDL-C, and drinking status, except for the stratification variable. Interaction terms between TyG and stratification variables were tested. ORs with 95% CIs were reported. ROC curve analysis was used to assess the discriminative ability of the TyG index and other lipid markers for SA. The optimal cut-off values were identified using the maximum Youden index. A two-sided *P* value < 0.05 was considered statistically significant.

## Results

### Baseline characteristics

The 13,473 participants were categorized into TyG tertiles (T1–T3). With increasing TyG tertiles, participants were older and had higher body weight, BMI, SBP, and DBP, as well as greater proportions of males, smokers, and drinkers (all *P* < 0.001). Biochemical profiles showed progressive increases in FPG, UA, TC, LDL-C, TG, and inflammatory markers (WBC count, neutrophils, lymphocytes, and hs-CRP), while HDL-C levels decreased across tertiles (all *P* < 0.001). The prevalence of SA increased markedly from 24.5% in T1 to 54.4% in T3 (*P* < 0.001) (Table 1). While mean SBP showed a positive correlation with TyG levels, the distribution of diagnosed hypertension varied across tertiles, with the T3 group showing a slightly lower frequency of HTN (23.5% vs. 27.4% in T1).

**Table 1:** Baseline characteristics of study participants according to TyG Index tertiles.

Variables	T1 (*n* = 4,491, ≤ 8.28)	T2 (*n* = 4,491, 8.28 < TyG ≤ 8.78)	T3 (*n* = 4,491, TyG > 8.78)	*P*-value
TyG index	7.97 (7.97–7.98)	8.53 (8.52–8.53)	9.25 (9.24–9.27)	< 0.001
Demographics				
Male, *n* (%)	957 (21.3%)	1,811 (40.3%)	2,513 (56.0%)	< 0.001
Age (years)	44.72 ± 10.25	49.76 ± 11.53	51.74 ± 11.46	< 0.001
Height (cm)	164.40 ± 6.97	165.59 ± 7.99	167.20 ± 8.66	< 0.001
Weight (kg)	62.00 ± 10.37	68.53 ± 12.16	74.80 ± 13.24	< 0.001
BMI (kg/m2)	22.87 ± 3.02	24.89 ± 3.34	26.63 ± 3.40	< 0.001
Lifestyles				
Smoking, *n* (%)	288 (6.4%)	604 (13.4%)	1,076 (24.0%)	< 0.001
Drinking, *n* (%)	548 (12.2%)	1,158 (25.8%)	1,763 (39.3%)	< 0.001
Clinical Measures				
SBP (mmHg)	118.28 ± 15.61	126.35 ± 17.66	132.61 ± 18.05	< 0.001
DBP (mmHg)	72.21 ± 10.50	77.28 ± 11.45	81.57 ± 11.88	< 0.001
FPG (mmol/L)	4.88[ 4.60,5.14]	5.12[4.82,5.50]	5.53[5.07,6.52]	< 0.001
UA (*μ*mol/L)	276.3 ± 70.4	317.2 ± 81.6	358.0 ± 89.3	< 0.001
Lipid Profile				
TG (mmol/L)^a^	0.76 [0.64, 0.87]	1.20 [1.07, 1.37]	2.02 [1.69, 2.62]	< 0.001
TC (mmol/L)	4.61 ± 0.84	4.92 ± 0.92	5.21 ± 1.07	< 0.001
HDL-C (mmol/L)	1.53 ± 0.30	1.41 ± 0.29	1.28 ± 0.27	< 0.001
LDL-C (mmol/L)	2.63 ± 0.72	2.98 ± 0.80	3.16 ± 0.90	< 0.001
Inflammation/Blood				
WBC (×10^9^/L)^a^	5.19 [4.43, 6.12]	5.71 [4.86, 6.67]	6.15 [5.26, 7.18]	< 0.001
Neutrophil^a^	2.87 [2.31, 3.59]	3.19 [2.59, 3.91]	3.47 [2.83, 4.20]	< 0.001
Lymphocyte^a^	1.74 [1.45, 2.07]	1.87 [1.54, 2.26]	2.02 [1.67, 2.41]	< 0.001
hs-CRP (mg/L)^a^	1.56 [1.20, 2.16]	1.82 [1.31, 2.74]	2.19 [1.50, 3.48]	< 0.001
Clinical History, n (%)				
HTN, *n* (%)	1,229 (27.4%)	1,152 (25.7%)	1,057 (23.5%)	< 0.001
HLP, *n* (%)	34 (0.8%)	38 (0.8%)	32 (0.7%)	0.762
DM, *n* (%)	561 (12.5%)	570 (12.7%)	661 (14.7%)	0.003
Outcome				
SA, *n* (%)	1,099 (24.5%)	1,910 (42.5%)	2,443 (54.4%)	< 0.001

Data for TyG index are presented as mean (95% confidence interval). Data are expressed as mean ± standard deviation (SD), median [Q, Q3], or n (%), as appropriate. Asterisks (^a^) denote variables with a non-normal distribution. BMI, body mass index; SBP, systolic blood pressure; DBP, diastolic blood pressure; FPG, Fasting Plasma Glucose; UA, Uric acid; TG, triglycerides; TC, total cholesterol; HDL-C, high density lipoprotein cholesterol; LDL-C, low density lipoprotein cholesterol; WBC, White blood cell; hs-CRP, High-sensitivity C-reactive protein; HTN, hypertension; DM, diabetes mellitus; HLP, hyperlipidemia.

### Association between TyG index and SA

Multivariable logistic regression analyses demonstrated a significant association between the TyG index and SA (Table 2). When analyzed as a continuous variable, each 1-unit increase in the TyG index was associated with a higher odds of SA in the fully adjusted model (OR = 1.20, 95% CI: 1.10–1.30, *P* < 0.001). When categorized into tertiles, a clear dose–response relationship was observed. Compared with participants in the lowest tertile (T1), those in T2 and T3 exhibited significantly higher odds of SA, with fully adjusted ORs of 1.19 (95% CI: 1.06–1.34) and 1.38 (95% CI: 1.22–1.56), respectively (*P_trend_* < 0.001) (Table 2).

**Table 2:** Association between the TyG index and SA in different models.

Exposure of TyG index	Model 1 (Crude)	Model 2 (Adjusted)	Model 3 (Adjusted)
Continuous variable			
Per 1-unit increase	2.33 (2.19–2.49)	1.41 (1.31–1.52)	1.20 (1.10–1.30)
*P* value	< 0.001	< 0.001	< 0.001
TyG tertiles			
Tertile 1 (Ref.)	1.00 (Reference)	1.00 (Reference)	1.00 (Reference)
Tertile 2	2.28 (2.09–2.50)	1.33 (1.19–1.48)	1.19 (1.06–1.34)
Tertile 3	3.68 (3.37–4.03)	1.71 (1.53–1.91)	1.38 (1.22–1.56)
*P* for trend	< 0.001	< 0.001	< 0.001
As threshold (RCS-derived)			
≤ 8.07 (Ref.)	1.00 (Reference)	1.00 (Reference)	1.00 (Reference)
> 8.07	2.89 (2.57–3.24)	1.34 (1.16–1.55)	1.20 (1.03–1.39)
*P* value	< 0.001	< 0.001	0.018

Model 1: unadjusted. Model 2: adjusted for age and sex. Model 3: additionally adjusted for BMI, smoking, SBP, HDL-C and drinking. P for trend was calculated by modeling the median value of each TyG tertile as a continuous variable.

No significant multicollinearity was observed among variables included in the model (all VIF values < 2.5; range: 1.31–2.36, Supplementary Table S2).

Additional analyses showed that the association remained significant after adjustment for LDL-C alone (OR = 1.12, 95% CI: 1.03–1.22, *P* = 0.006), but was attenuated when LDL-C and HDL-C were included simultaneously (OR = 1.03, 95% CI: 0.94–1.12, *P* = 0.572) (Supplementary Table S3). Standardized estimates indicated that LDL-C showed a stronger association with SA (standardized OR = 1.33, 95% CI: 1.27–1.40) compared with TyG (standardized OR = 1.12, 95% CI: 1.06–1.17) (Supplementary Table S4). These comparative associations were further visualized using RCS analysis based on standardized Z-scores (Supplementary Figure S1).

### Non-linear association and sensitivity analysis

**Figure 2:**
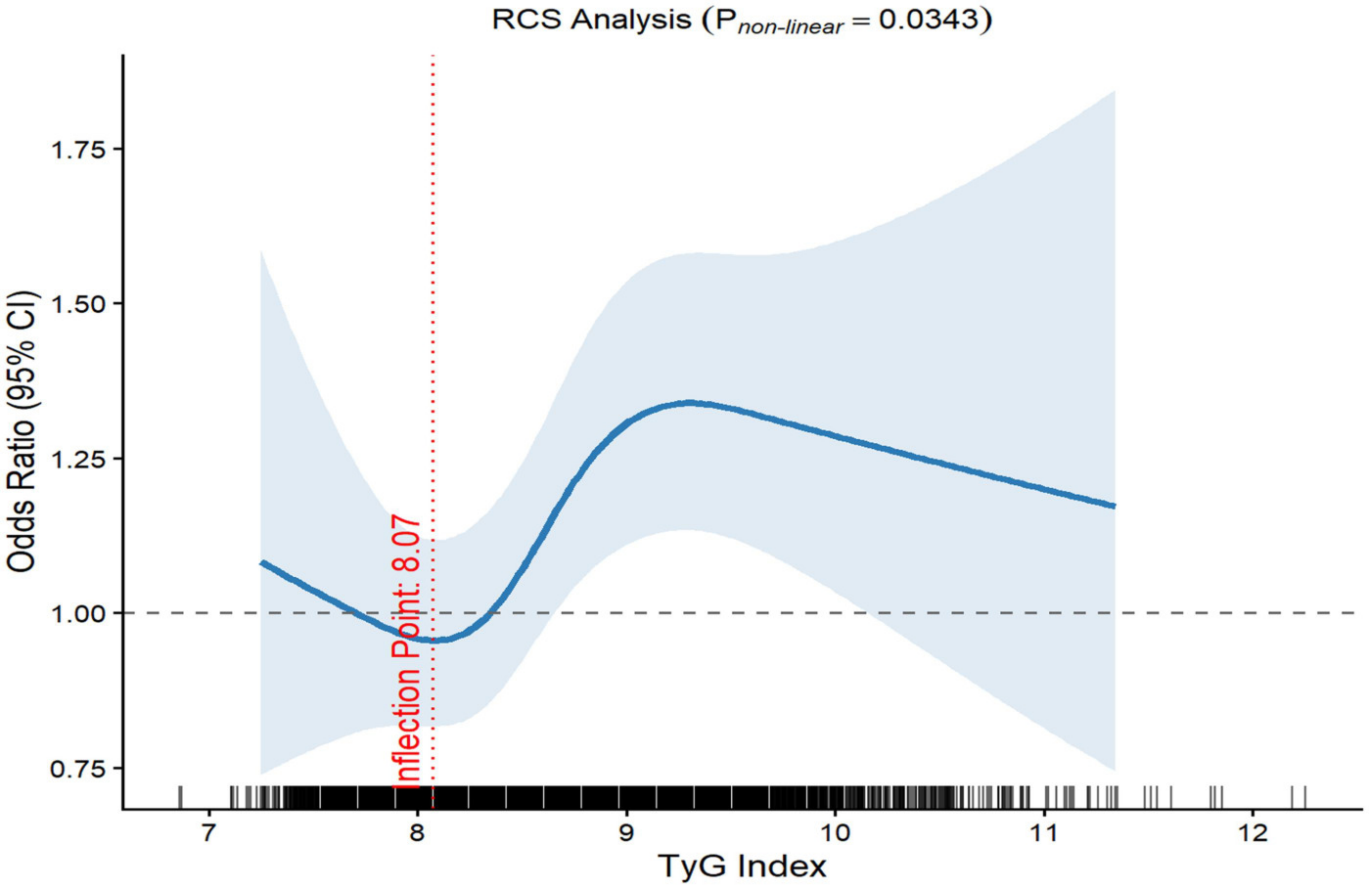
RCS analysis of the association between the TyG index and SA risk. A potential non-linear association was observed (*P*_*non-linear*_ = 0.0343), with an apparent model-derived inflection point around 8.07. The model was adjusted for age, sex, BMI, smoking, drinking, SBP, HDL-C. The solid line represents the estimated ORs, and the shaded area indicates the 95% CIs. The curve at the extreme ranges should be interpreted with caution due to limited data density.

RCS analysis revealed a significant non-linear relationship between the TyG index and SA (*P_non−linearity_*=0.0343) (Figure 2). A distinct inflection point was identified at a TyG value of 8.07. Below this point, SA risk remained relatively stable, whereas above it, the risk increased sharply. Further categorized analysis based on this inflection point showed that participants with a TyG index > 8.07 had a significantly higher odds of SA compared with those with TyG ≤ 8.07 in the fully adjusted model (OR=1.20, 95% CI: 1.03–1.39, *P* = 0.018).

After excluding extreme TyG values (below 2.5th and above 97.5th percentiles), the association remained significant, though the non-linear evidence was attenuated (*P_non−linearity_*=0.2261). Within the restricted TyG range of 7.63–9.94, the curve showed an approximately monotonic pattern (Supplementary Figure S2), and the dose-response relationship across tertiles remained consistent (*P_trend_* < 0.001).

### Subgroup analyses

**Figure 3:**
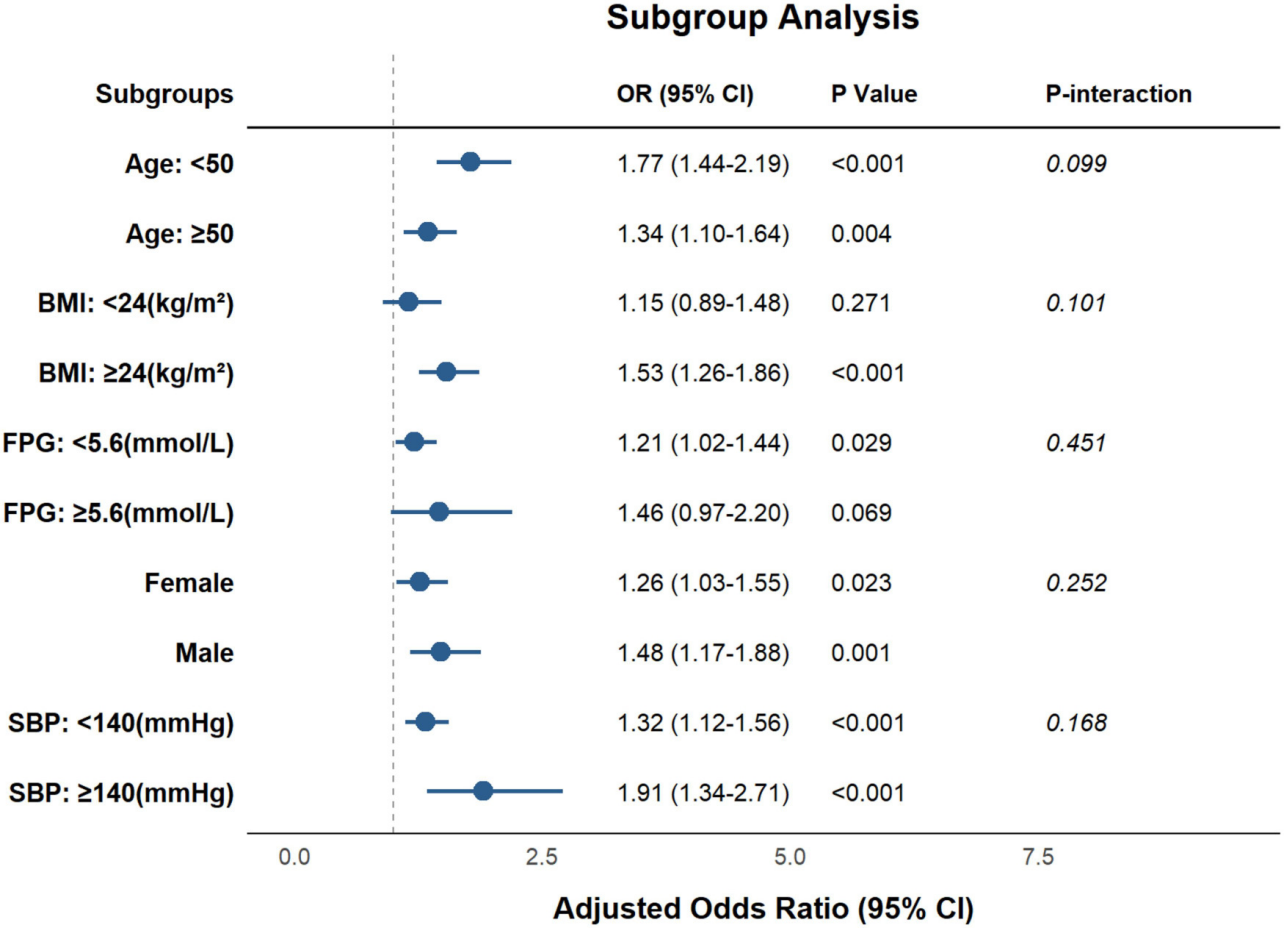
Subgroup analysis of the association between the TyG index and SA. Forest plot showing the OR (95% CI) for SA, comparing the highest TyG tertile (T3) with the lowest tertile (T1). Subgroup analyses were adjusted for age, sex, BMI, smoking, drinking, SBP, and HDL-C, except for the stratification variable. The vertical dashed line represents the reference (OR = 1.0). *P* value represents the significance within each subgroup, and P-interaction indicates the statistical significance of the interaction between TyG index and the stratification variables.

Subgroup analyses demonstrated that the association between the TyG index and SA risk remained consistent and significant across both sexes, age groups, and SBP categories, without significant interactions (all *P_interaction_*> 0.05) (Figure 3). The effect of the TyG index appeared more pronounced in overweight/obese individuals (OR = 1.53, 95%CI: 1.26–1.86) than in those with normal weight, although the interaction was not statistically significant (*P* = 0.101). Notably, the TyG index remained significantly associated with SA among normoglycemic individuals (FPG < 5.6 mmol/L; OR=1.21, 95%CI: 1.02–1.44). Although the association in the elevated glucose group (FPG≥5.6 mmol/L) did not reach statistical significance (*P* = 0.072), possibly due to a relatively smaller sample size, the higher point estimate (OR=1.46) suggests a possible trend toward a stronger association.

### ROC analysis for the discriminative ability of TyG Index

**Figure 4:**
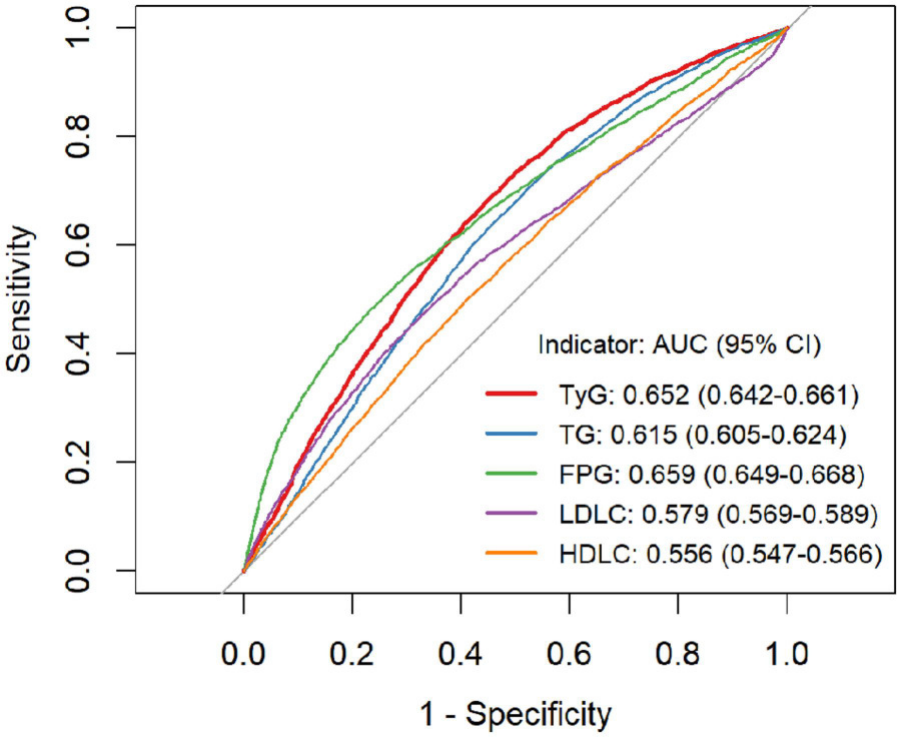
ROC curves of the TyG index and metabolic parameters in assessing their value for identifying SA.

ROC analysis showed that the TyG index had a moderate discriminative ability for SA (Figure 4). The AUCs and optimal clinical thresholds for all parameters are detailed in Table 3.

**Table 3:** ROC analysis of TyG and metabolic parameters for SA.

Parameter	AUC (95% CI)	Cut-off Value	Sensitivity	Specificity
TyG Index	0.652 (0.642–0.661)	8.445	0.686	0.549
FPG (mmol/L)	0.659 (0.649–0.668)	5.385	0.48	0.768
TG (mmol/L)	0.615 (0.605–0.624)	1.135	0.644	0.538
LDL-C (mmol/L)	0.579 (0.569–0.589)	3.115	0.473	0.673
HDL-C (mmol/L)	0.556 (0.547–0.566)	1.345	0.506	0.583

The optimal cut-off values were determined using the maximum Youden index (Sensitivity+Specificity - 1). For HDL-C, the direction of the ROC curve was set to indicate that lower values are associated with a higher risk of subclinical atherosclerosis, whereas for all other parameters, higher values indicate higher risk.

A potential non-linear association was observed (*P*_non−linea*r*_ = 0.0343), with an apparent model-derived inflection point around 8.07. The model was adjusted for age, sex, BMI, smoking, drinking, SBP, HDL-C. The solid line represents the estimated ORs, and the shaded area indicates the 95% CIs. The curve at the extreme ranges should be interpreted with caution due to limited data density.

Forest plot showing the OR (95% CI) for SA, comparing the highest TyG tertile (T3) with the lowest tertile (T1). Subgroup analyses were adjusted for age, sex, BMI, smoking, drinking, SBP, and HDL-C, except for the stratification variable. The vertical dashed line represents the reference (OR = 1.0). *P* value represents the significance within each subgroup, and P-interaction indicates the statistical significance of the interaction between TyG index and the stratification variables.

## Discussion

This large-scale study in an asymptomatic population provides a comprehensive evaluation of the association between the TyG index and SA. Our findings indicated a positive association between TyG index levels and the odds of SA, with evidence of a potential non-linear pattern. Notably, this association remained significant among normoglycemic individuals, suggesting that the TyG index may capture early metabolic alterations beyond conventional glucose measures.

In parallel with increasing TyG levels, we observed progressive elevations in BMI, SBP, DBP, TC, LDL-C, as well as inflammatory markers such as WBC count and hs-CRP. These findings indicate that the TyG index reflects the clustering of metabolic and inflammatory abnormalities, rather than isolated glycemic or lipid disturbances. Consistent with previous studies (5, 9, 18), our results support the role of the TyG index as an integrative marker of global metabolic dysregulation and early vascular injury in asymptomatic populations.

Biologically, the TyG index reflects the combined effects of hypertriglyceridemia and hyperglycemia, both closely linked to IR. IR has been linked to reduced nitric oxide bioavailability (19), increased oxidative stress, vascular smooth muscle cell proliferation, and enhanced macrophage lipid uptake (4, 19, 20), all of which are associated with early atherogenic processes. The concomitant increase in inflammatory biomarkers observed in our study further supports the close interconnection between metabolic and inflammatory pathways in the initiation of atherosclerosis. These findings are consistent with the metabolic–inflammatory framework proposed by Hotamisligil and the modern inflammatory paradigm of atherosclerosis described by Libby (21, 22). In addition, recent reviews have emphasized that the TyG index may serve as a practical surrogate marker reflecting the interaction between IR, chronic low-grade inflammation, and vascular injury (5, 10).

Multivariable logistic regression analyses further suggested that the TyG index was independently associated with SA. Even after full adjustment, each one-unit increase in TyG was associated with approximately 20% higher odds of SA, and multicollinearity diagnostics showed low VIF values across all covariates, ensuring the robustness of these findings. In the context of this large-scale cohort, such an increase suggests that even modest elevations in the TyG index could translate into a substantial burden of subclinical disease at the population level. However, standardized analyses revealed that LDL-C exhibited a stronger association with SA compared to the TyG index. This aligns with the established paradigm that cumulative, long-term LDL-C exposure directly drives atherosclerosis development (23, 24). Furthermore, the association between TyG and SA was partially attenuated after simultaneous adjustment for LDL-C and HDL-C, indicating an overlap in the metabolic pathways captured by these markers. Despite this partial overlap, the TyG index distinctly reflects broader metabolic dysfunctions—specifically insulin resistance—that extend beyond pure cholesterol-mediated damage. Therefore, rather than serving as a primary determinant of atherosclerosis, the TyG index provides valuable complementary information and functions as a practical adjunct marker for evaluating residual metabolic risk. This robust positive association aligns with a previous secondary analysis of a Japanese national cross-sectional study, which also demonstrated that higher TyG levels were independently linked to an increased risk of atherosclerosis after adjusting for multiple confounders (25). Supporting this, a large meta-analysis confirmed that a higher TyG index independently predicts increased risks of SA and arterial stiffness across various populations (26).

In contrast to studies where the association attenuated after full adjustment (27), the persistence of significance in our study may be attributed to the larger sample size, greater statistical power, and the use of a sensitive subclinical endpoint. Collectively, these findings support the potential role of TyG as a continuous marker associated with cardiovascular risk.

In the primary analysis, we observed a statistically significant non-linear association between the TyG index and SA, with an apparent model-derived inflection point around 8.07. In sensitivity analyses excluding extreme TyG values, the previously observed non-linear pattern was attenuated, suggesting that the curvature at the tails may be influenced by sparse data and increased variability in extreme TyG ranges rather than a robust biological effect. As shown in Supplementary Figure S2, OR estimates below 1.0 on the left side of the reference value should be interpreted as relatively lower odds compared with the median TyG level, rather than evidence of a protective effect. Overall, the association between TyG and SA remained generally positive across the observed range. Therefore, the observed non-linear relationship and the identified inflection point should be regarded as exploratory and warrant further validation in prospective studies.

Our findings are broadly consistent with previous studies examining the association between the TyG index and carotid atherosclerosis (28, 29). Liu et al. (30) reported a non-linear relationship between TyG levels and carotid plaque in a Japanese check-up population (inflection point 9.06). Wang et al. (31) observed that the TyG index was independently associated with unstable carotid plaque characteristics in nondiabetic adults, and Wu et al. (32) further showed that higher TyG levels predicted incident carotid atherosclerosis in a prospective Chinese cohort. Similarly, Yang et al. (25) and Guo et al. (33) also reported positive associations between TyG and carotid atherosclerosis in large cross-sectional populations. Compared with these studies, our findings extend the existing evidence by focusing on a large asymptomatic health check-up population and by demonstrating that the association between TyG and SA persists even among individuals without overt dysglycemia.

Importantly, although the absolute inflection point identified in our study was lower than that reported by Liu et al. (30), the overall direction of association remained consistent across studies. Differences in population characteristics, metabolic status, ethnicity, sample size, and the distribution of TyG values may partly explain these discrepancies.

In addition, our subgroup analyses demonstrated that the association between the TyG index and SA remained significant even in normoglycemic individuals, suggesting that the TyG index may capture early metabolic derangements and vascular abnormalities beyond conventional glucose measures. This finding is consistent with previous studies indicating that the TyG index can identify “hidden” metabolic risks even among individuals without overt diabetes or traditional metabolic abnormalities (18). While the association did not reach statistical significance in the elevated FPG group (*P* = 0.072), the point estimate remained higher, suggesting a possible trend that warrants cautious interpretation and further validation in larger samples.

Subgroup analyses further suggested the broad applicability of the TyG index across several clinically relevant strata. The association appeared more pronounced among males, overweight or obese individuals, and participants with elevated SBP, suggesting that metabolic and hemodynamic factors may jointly contribute to vascular vulnerability. However, the association was not statistically significant in the normal-BMI subgroup, suggesting that adiposity may modify the relationship between TyG and SA and that the discriminative ability of TyG index may be enhanced when combined with anthropometric indicators. This is supported by a recent nationwide prospective study showing that integrating the TyG index with body shape indicators significantly improves the prediction of vascular events (34). Further mechanistic studies are warranted to clarify these divergent subgroup effects.

Subgroup findings were generally consistent but should be interpreted cautiously, as these analyses were exploratory, involved multiple comparisons, and show largely non-significant interactions, potentially increasing the risk of type I error.

Interestingly, while SBP progressively increased across TyG tertiles, the prevalence of hypertension was higher in the lowest tertile. This discrepancy may reflect the difference between historical diagnosis and single time-point blood pressure measurements. In the present study, hypertension was defined as a composite of prior diagnosis, antihypertensive medication use, or elevated blood pressure at examination. Therefore, the higher prevalence in the lowest tertile may be driven by a larger proportion of previously diagnosed patients whose blood pressure is strictly controlled by medication. Conversely, individuals in the higher TyG strata may experience progressively rising blood pressure driven by metabolic dysfunction, but many may remain undiagnosed or have not yet crossed the diagnostic threshold.

To further evaluate its clinical utility, ROC analysis indicated that the TyG index possesses a moderate discriminative capacity for SA, comparable to traditional lipid markers. The identified threshold provides a quantitative reference for its use as a cost-effective screening tool during routine health check-ups, particularly for identifying hidden subclinical vascular injury in normoglycemic individuals. This aligns with recent meta-analytic and longitudinal evidence supporting the stable association of TyG with atherosclerosis across diverse populations and the clinical importance of long-term metabolic monitoring (26, 35). Overall, the TyG index may be more relevant as an adjunctive marker for population-level vascular assessment rather than as a standalone diagnostic tool.

Several limitations should be acknowledged. First, the cross-sectional design precludes causal inference regarding the association between the TyG index and SA. Second, residual confounding cannot be entirely excluded. Although we adjusted for several conventional risk factors, information regarding medication use, physical activity, and dietary habits, was unavailable and may have influenced the observed associations. Third, the TyG index was calculated from single time-point fasting measurements, which may not adequately represent long-term metabolic exposure. Although most variables were comparable between groups, mild to moderate imbalances in sex and age (SMD ranging from 0.187 to 0.261) were observed, which may indicate potential selection bias. In addition, formal inter- and intra-observer reproducibility analyses for carotid ultrasonography were not performed, although standardized scanning protocols and quality control procedures were implemented to minimize measurement variability. Fourth, this was a single-center study based on a health check-up population, which may introduce healthy volunteer bias and limit generalizability to broader or higher-risk populations. Finally, although the RCS analysis suggested a potential non-linear relationship, the curve shape at extreme TyG values should be interpreted cautiously because it may be affected by sparse observations and model instability.

Therefore, future multi-center and prospective studies in more diverse populations are warranted to validate these findings.

## Conclusion

In summary, our findings suggest that the TyG index is associated with IR, inflammatory status, and subclinical vascular changes in an asymptomatic population. The observed dose–response relationship and generally consistent associations across clinically relevant subgroups support its potential relevance as a metabolic marker linked to early atherosclerotic changes. Although the identified non-linear inflection point provides a quantitative reference, future longitudinal and interventional studies are warranted to validate its clinical utility and incremental value in cardiovascular risk assessment.

## Data Availability

The original contributions presented in the study are included in the article/Supplementary Material, further inquiries can be directed to the corresponding author/s.
