# Supplementary material for: Association between the triglyceride–glucose index and subclinical atherosclerosis in asymptomatic adults: a large cross-sectional study of a health check-up population
Source: Front Cardiovasc Med. 2026 Jun 10;13:1795702. doi: 10.3389/fcvm.2026.1795702 (PMC13290593; doi:10.3389/fcvm.2026.1795702)
Supplement: Supplementary file 1 [file Datasheet1.pdf]

Supplementary Materials

Supplementary Table

Table S1. Comparison of baseline characteristics between included and excluded participants

Characteristic	Excluded Group	Included Group (N=13,473)	P value	SMD
Age(years)	45.33 (14.41) (n=145,672)	48.74 (11.48)	< 0.001	0.261
			< 0.001	
Sex(n,%)				
			< 0.001	
Male(n,%)	70580(48.45)	5281(39.20)		0.187
Female	75092(51.55)	8192(60.80)		
BMI (kg/m^2)	24.89 (3.79) (n=127,040)	24.80 (3.60)	0.011	0.023
SBP (mmHg)	127.01 (18.29) (n=127,059)	125.75 (18.12)	< 0.001	0.069
DBP (mmHg)	77.74 (12.06) (n=127,059)	77.02 (11.92)	< 0.001	0.06
FPG (mmol/L)	5.35 (1.28) (n=131,433)	5.46 (1.41)	< 0.001	0.082
TG (mmol/L)	1.51 (1.15) (n=129,172)	1.46 (1.12)	< 0.001	0.04
LDL-C(mmol/L)	2.88 (0.84) (n=127,078)	2.92 (0.84)	< 0.001	0.048
HDL-C(mmol/L)	1.38 (0.30) (n=127,078)	1.40 (0.30)	< 0.001	0.091

Note: Values are presented as mean (standard deviation) or number (percentage), as appropriate. P values were calculated using the Student's t-test or Mann–Whitney U test for continuous variables, as appropriate based on data distribution, and the chi-square test for categorical variables. Standardized mean differences (SMDs) were used to assess the magnitude of differences between groups, with SMD < 0.1 generally indicating negligible imbalance.

The number of participants (n) varies across characteristics in the excluded group due to missing values in the original health examination records. Standardized mean differences (SMD) were calculated based on available data for each variable.

Despite statistically significant P values driven by the large sample size, most SMDs

were small, indicating overall comparability between groups.

Table S2. Assessment of multicollinearity using VIF

Variables	VIF
TyG index	1.43
Age	1.31
Sex (Male)	2.36
BMI	1.42
SBP	1.46
HDL-C	1.34
Smoking status	1.49
Drinking status	2.28

Note: All VIF values were below 2.5, indicating no significant multicollinearity.

Table S3. Association between TyG index and SA under different lipid-adjusted models

Models	Lipid Adjustment	Odds Ratio (95% CI)	P value
Model 3	Adjusted for HDL-C, without LDL-C	1.20 (1.10–1.30)	< 0.001

Models	Lipid Adjustment	Odds Ratio (95% CI)	P value
Model 3.5	Adjusted for LDL-C, without HDL-C	1.12 (1.03–1.22)	0.006
Model 4	Adjusted for both HDL-C and LDL-C	1.03 (0.94–1.12)	0.572

Note: Data are presented as ORs and 95% CIs. All models were adjusted for age, sex, BMI, smoking status, SBP, and drinking status. Model 3 included HDL-C but not LDL-C; Model 3.5 included LDL-C but not HDL-C; Model 4 included both HDL-C and LDL-C simultaneously.

Table S4. Association Between Standardized TyG Index, LDL-C, and SA.

Variable	Standardized OR (95% CI)	P Value
TyG index	1.12 (1.06–1.17)	< 0.001
LDL-C	1.33 (1.27–1.40)	< 0.001

Note: ORs were calculated per 1- SD increase in each variable to allow for direct comparison between different indicators. The model was adjusted for age, gender, and other potential confounders.

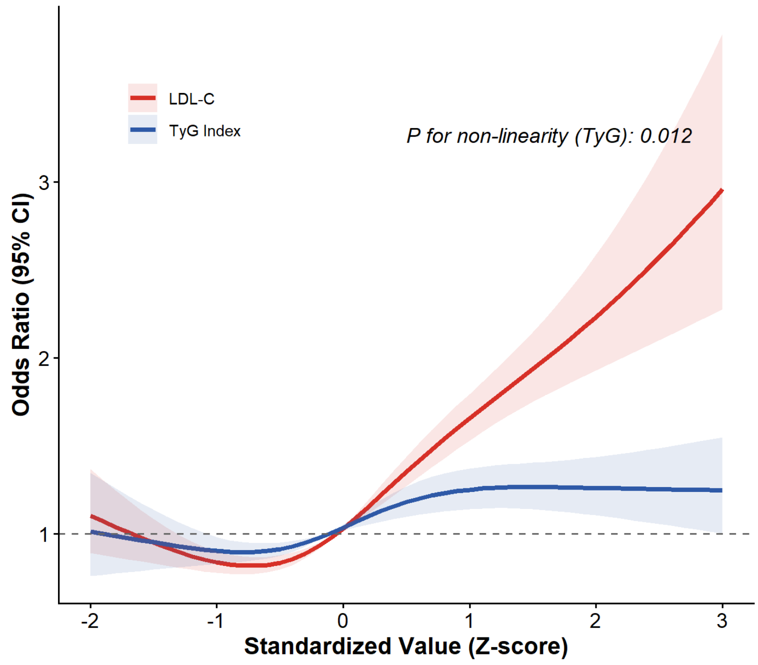

Figure S1. Dose-response association of TyG index and LDL-C with SA risk.

Standardized RCS curves show the association of TyG index (blue) and LDL-C (red) with SA risk. Shaded areas represent 95% CIs. All models were adjusted for covariates in Model 3. $P_{non\text{-}linearity}$ (TyG) = 0.012.

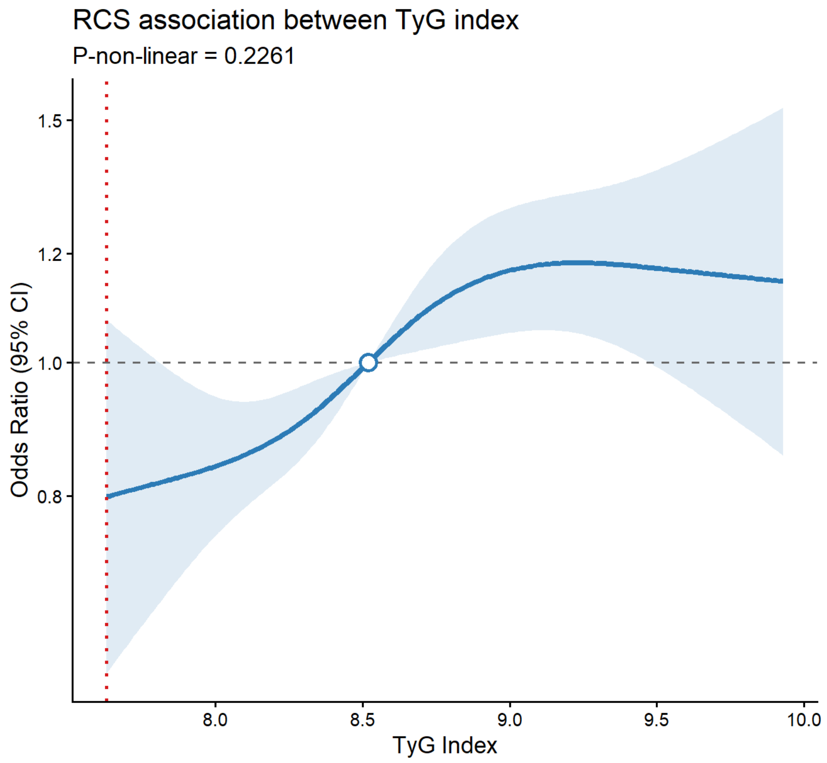

Figure S2.

Sensitivity analysis of the association between TyG index and SA. RCS analysis was performed after excluding extreme TyG values (below the 2.5th and above the 97.5th percentiles). The solid blue line represents the estimated ORs, and the light blue shaded area represents the 95% CIs. ORs were calculated relative to the median TyG value, which was used as the reference point (OR = 1.0, indicated by the open circle). OR estimates below 1.0 on the left side of the reference point indicate relatively lower odds compared with the reference value rather than a protective effect. The model was adjusted for age, sex, BMI, smoking, drinking, SBP, HDL-C. The horizontal dashed line indicates the reference OR of 1.0. The p-value for non-linearity was 0.2261.

Abbreviations:

TyG, triglyceride–glucose index;

SA, subclinical atherosclerosis;

BMI， body mass index;

SBP, systolic blood pressure;

HDL-C, high-density lipoprotein cholesterol;

LDL-C, low-density lipoprotein cholesterol;

FPG, fasting plasma glucose;

TG, triglycerides;

VIF, Variance Inflation Factor;

RCS, restricted cubic spline;

OR, odds ratio;

CI, confidence interval.
